# Doxorubicin Activates Hepatitis B Virus Replication by Elevation of p21 (Waf1/Cip1) and C/EBPα Expression

**DOI:** 10.1371/journal.pone.0131743

**Published:** 2015-06-29

**Authors:** Yu-Fang Chen, Chin-Liew Chong, Yi-Chieh Wu, Yi-Ling Wang, Kuen-Nan Tsai, Tzer-Min Kuo, Ming-Hsiang Hong, Cheng-po Hu, Mong-Liang Chen, Yu-Chi Chou, Chungming Chang

**Affiliations:** 1 Institute of Microbiology and Immunology, National Yang-Ming University, Taipei, Taiwan; 2 Institute of Molecular and Genomic Medicine, National Health Research Institutes, Miaoli, Taiwan; 3 Faculty of Traditional Chinese Medicine, Southern University College, Johor Bahru, Malaysia; 4 Institute of Molecular Medicine, National Tsing Hua University, Hsinchu, Taiwan; 5 Graduate Institute of Clinical Medical Science, China Medical University, Taichung, Taiwan; 6 Institute of Biomedical Sciences, Academia Sinica, Taipei, Taiwan; 7 Department of Life Science, Tunghai University, Taichung, Taiwan; 8 Center for Molecular Medicine, China Medical University and Hospital, Taichung, Taiwan; 9 Institute of Molecular Biology, Academia Sinica, Taipei, Taiwan; Kaohsiung Medical University Hospital, Kaohsiung Medical University, TAIWAN

## Abstract

Hepatitis B virus reactivation is an important medical issue in cancer patients who undergo systemic chemotherapy. Up to half of CHB carriers receiving chemotherapy develop hepatitis and among these cases a notable proportion are associated with HBV reactivation. However, the molecular mechanism(s) through which various chemotherapeutic agents induce HBV reactivation is not yet fully understood. In this study, we investigated the role of the cell cycle regulator p21 (Waf1/Cip1) in the modulation of HBV replication when a common chemotherapeutic agent, doxorubicin, is present. We showed that p21 expression was increased by doxorubicin treatment. This elevation in p21 expression enhanced the expression of CCAAT/enhancer-binding protein α (C/EBPα); such an increase is likely to promote the binding of C/EBPα to the HBV promoter, which will contribute to the activation of HBV replication. Our current study thus reveals the mechanism underlying doxorubicin modulation of HBV replication and provides an increased understanding of HBV reactivation in CHB patients who are receiving systemic chemotherapy.

## Introduction

Human hepatitis B virus (HBV) is a small DNA virus that infects hepatocytes and infection can lead to various liver pathological changes, including acute self-limited hepatitis, fulminant hepatitis, chronic hepatitis, liver cirrhosis, and hepatocellular carcinoma (HCC) [1]. Despite preventive vaccines and therapeutic agents that act against HBV being available for decades, HBV infection remains a serious global public health issue, with 350 million chronic hepatitis B (CHB) carriers worldwide [2]. Up to 25% of CHB patients ultimately develop cirrhosis or HCC [3], of which about 12% eventually die of liver failure (due to cirrhosis) and 10% die from liver cancer [4, 5]. The number of annual deaths attributed to cirrhosis and HCC is over 1 million [6, 7].

Doxorubicin, a FDA-approved chemotherapeutic agent, is used in the treatment of a wide range of cancers, including leukemias, lymphoma, bladder cancer, breast cancer, soft tissue sarcoma, multiple myeloma, and others. However, cancer patients receiving chemotherapy, including doxorubicin treatment, are found to be at high risk of HBV reactivation, which is a common cause of liver damage during chemotherapy in chronic HBV-infected patients [8–11]. The frequency of HBV reactivation is estimated to be 20% to 36% in CHB patients who undergo chemotherapy and is estimated to account for up to 44% to 58% of the incidence of hepatitis [10, 11]. A prospective study of HBV reactivation by chemotherapy revealed that the risk factors include male sex, younger age, HBeAg positivity, and a diagnosis of lymphoma [10]. Moreover, an *in vitro* study of 2.2.15 cells showed a dramatic increase in HBV replication after doxorubicin treatment, which suggests that there is direct stimulation of viral replication, rather than indirect immunosuppression, and this is what triggers HBV reactivation during chemotherapy [12]. However, the exact mechanism through which chemotherapeutic agents, including doxorubicin, trigger HBV reactivation has not yet been explored.

Many chemotherapeutic agents have been reported to induce DNA damage and activate the expression of p53 as well as p21 (Waf1/Cip1), leading to cell cycle arrest [13, 14]. Several studies have indicated that the replication of HBV is highly correlated with the cell cycle of hepatocytes and that HBV replication is remarkably activate during the G0/G1 phase [15, 16]. Our previous study also has shown that the number of viral replicative intermediates is significantly increased after cells reached confluence [17]. These findings suggest that HBV replication is highly dependent on the growth status of hepatocytes. The fact that chemotherapeutic agents reactivate HBV replication is therefore highly likely to be due to these chemicals being involved in the manipulation of cell cycle progression.

The cell cycle regulator p21 has been previously identified to be directly involved in the regulation of cyclin-dependent kinase (CDK) and to induce cell cycle G0/G1 arrest. Increasing evidence suggests that p21 is not only a cell cycle blocker, but also a multifunctional protein that directly modulates the transcription program by interfering with transcription factor complex assembly [18, 19]. For instance, p21 has been shown to be able to bind to c-Myc and block the formation of the c-Myc-Max complex, thus modulating the transcriptional activation function of this complex [19]. An increase in the expression level of p21 has been found to inhibit E2F-mediated transcription by disrupting the cdk2-containing E2F-p130 complex [18]. In addition, p21 is able to interact with a range of transcription factors, such as CCAAT/enhancer-binding protein α (C/EBPα) [20], and bring about a modulation of their biological functioning. The effect of the expression level of p21 in relation to its interaction with transcription factors, as well as this protein's involvement in HBV replication, remains unclear.

In the current study, we investigated the underlying mechanism through which doxorubicin treatment activates HBV replication; to do this we used a well characterized HBV-producing cell line, 1.3.ES2 cells. The roles of p21 and C/EBPα, in doxorubicin-mediated HBV activation were explored. Our study provides new insights that help us to understand HBV reactivation when it is induced by a chemotherapeutic agent.

## Materials and Methods

### Cell culture

The 1.3.ES2 cell line is derived from HepG2 cells and contains one integrated copy of a 1.3-fold HBV genome [21]. The 1.3.ES2 cells and HepG2 cells were propagated in Dulbecco’s modified Eagle’s medium (DMEM) (Gibco Laboratories, Grand Island, NY) supplemented with 10% fetal bovine serum, 100 IU/ml penicillin, 100 μg/ml streptomycin, 2 mM L-glutamine, and 100 μM non-essential amino acids. The cells were grown at 37°C in a 5% CO_2_ incubator.

### Plasmids

For the construction of the luciferase reporters, different HBV promoter regions (CP, XP, BCP, EnI, EnI plus CP, and CPD1) were amplified from the ayw subtype of the HBV genome [22] and then subcloned between the MluI and HindIII restriction sites of the pGL3-Basic luciferase vector (Promega Corporation, Madison, WI) [23]. The reporter pGL3/CPm, with two mutated C/EBP binding sites, was generated from pGL3/CP using the Quickchange II Site-Directed Mutagenesis Kit (Strategene). The wild-type C/EBP binding sites of CP, EnI and XP were substituted with following sequences:

CPm: GCTCCATAAG and TCAGTGCGAT

EnI-m1: CTATTGATTGGA

EnI-m2: AATTGTTTCGCC

EnI-m3: GCGAACTACCCC

XPm: GCGAACTACCCC

The p1.3HBcl/Hyg plasmid was derived from plasmid pHBV1.3. In this new plasmid, the transcription of the pgRNA was controlled by its own core promoter and the enhancer I and II regulatory elements [21]. pShIE was constructed by cloning into the multiple cloning site of pShuttle vector (CLONTECH) a fragment from the internal-ribosome-entry-site sequence; this was followed by an EGFP gene. The pShuttle/IE/p21 (pShIE/p21) plasmid was constructed by cloning a human p21 gene into the pShuttle/IE backbone. The pShuttle/1.3×HBV plasmid was constructed by cloning a 1.3-fold HBV genome (ayw subtype) [22] into a modified pShuttle vector backbone. pShuttle/1.3×HBV was ligated into Adeno-X viral DNA in order to produce Ad/1.3×HBV.

### DNA transfection

DNA transfection was performed according to the Lipofectamine 2000 Transfection Reagent (Invitrogen) protocol. In brief, 1.5 × 10^6^ cells were plated in 2 ml of the Dulbecco’s modified Eagle medium (DMEM) (Gibco Laboratories, Grand Island, NY) supplemented with 10% fetal bovine serum (FBS) per well of a 6-well plate one day before transfection. After overnight culture, the medium was replaced with 2 ml of serum-free DMEM medium 4 hours before transfection. Into each well, 5 μg of DNA was diluted into 250 μl OptiMEM I Medium. In the meantime, 5 μl of Lipofectamine 2000 reagent was diluted into 250 μl OptiMEM I Medium and incubated for 5 minutes at room temperature. After 5 minutes of incubation, the diluted lipofectamine reagent was combined with the diluted DNA and the mixture incubated for 20 minutes at room temperature. The DNA-Lipofectamine 2000 complexes were then added to each well and mixed gently by rocking the plate back and forth. This was followed by incubation for 16 hours at 37°C in a CO_2_ incubator. Finally the serum-free DMEM medium was replaced with the DMEM medium supplemented with 10% FBS.

### Recombinant adenovirus and infection

Adenovirus was prepared according to the BD Adeno-X Expression System 1 User Manual (Clonetech). In brief, a gene fragment was moved from the pShuttle vector into the pAdeno-X vector, and the pAdeno-X was transfected into HEK293 cells. Various recombinant adenoviruses carrying various genes, namely EGFP, p21, and 1.3× HBV separately, could be collected from the cells after several cycles of freeze and thawing. The viruses were then purified by centrifugation using the CsCl solution method. After this purification the various viruses were ready for use in cell infection experiments. After 2 hours of infection using HepG2 cells, the viruses were removed and fresh medium was added.

### Protein isolation and Western blot analysis

Cells were harvested to allow protein isolation using lysis buffer containing 1% Triton X-100 and protease inhibitor Complete (Roche, Mannheim, Germany). The cell lysate was then centrifuged at 15,500 × g for 15 min at 4°C, and the supernatant collected. Total protein was separated by SDS-15% polyacrylamide gel electrophoresis (PAGE) and transferred onto PVDF membranes (Millipore, Billerica, MA) using a Trans-Blot Semi-Dry Transfer Cell (Bio-Rad). The membrane was then blocked with 5% nonfat milk and probed with various antibodies as indicated. The immunoblot signal was detected using enhanced chemiluminescence reagent (PerkinElmer Life Sciences, Melbourne, Australia). The antibodies used in the study were anti-HBc antibody (Dako Cytomation, Glostrup, Denmark), anti-actin antibody (Sigma, St. Louis, MO), anti-P53 (Santa Cruz Biotechnology), anti-phospho-P53 (Santa Cruz Biotechnology), anti-P21(Calbiochem Biochemicals), anti-CEBP (GeneTex), anti-ERK (Merck Millipore), and anti-ERK-p (Cell Signaling Technology).

### Particle blot analysis

The intracellular HBV core particles were analyzed as previously described [24, 25]. Briefly, equal amounts of cell lysates were separated by separating on a 1.2% native agarose gel and transferred onto polyvinylidene fluoride membranes or nylon membrane in order to detect the HBV core capsid and capsid-associated nucleic acids respectively. The HBV core capsids were examined by immunoblot analysis using an anti-HBc antibody. The capsid-associated nucleic acids were released from the core particles *in situ* by treating the membranes with 0.2 N NaOH/1.5 M NaCl, which was followed by neutralization using 0.2 N Tris-HCl/1.5 M NaCl. Finally, the membranes were hybridized with a HBV-specific probe.

### RNA preparation

The cells were washed twice with cold GKNP. TRIzol Reagent (Invitrogen) was then added, and the samples were placed on ice for 10 min in order to lyse the cells. For each 1 ml of TRIzol Reagent, 200 μl of chloroform was added and the sample mixed by vortexing for 15 s in order to homogenize the samples. The samples were then centrifuged at 12,000 rpm at 4°C for 15 min. Following centrifugation, the aqueous phase was transferred to a fresh tube, and the RNA was precipitated by adding 600 μl of isopropanol. The RNA pellets were collected after centrifugation and then dissolved in diethyl-pyrocarbonate-treated water.

### Northern blot analysis

In total, 15 micrograms of total RNA were resolved electrophoretically on a 1.2% agarose gel with 2.2 M formaldehyde; this was followed by upward transfer to a nylon membrane overnight and cross-linking by UV irradiation. The 18S ribosomal RNA was used as the internal control for normalization. Prehybridization and hybridization were then performed in HYB-9 DNA hybridization solution (Gentra). After prehybridization at 65°C for 4 h, the indicated probes were added and hybridized for 12 h. The membrane was washed three times with 1 × SSC/0.1% SDS buffer at 52°C for 20–30 min each. The RNA was detected by autoradiography by exposure to X-ray film for 16 h at -80°C.

### Southern blot analysis

In total, 15 micrograms of total DNA was digested with HindIII and separated on a 0.8% agarose gel. After electrophoresis, the agarose gel was soaked in denaturing buffer (0.5 M NaOH, 1.5 M NaCl) twice for 15 min each time and then neutralized with neutralizing buffer (1M Tris-HCl [pH 8.0], 1.5 M NaCl). The DNA samples were then transferred to nylon membranes (Hybond-XL; Amersham Pharmacia Biotech) and UV cross-linked. The membranes were prehybridized at 42°C for 4 h in prehybridization solution and then hybridized in hybridization solution with a ^32^P-radiolabeled DNA probe (2 × 10^8^ cpm/μl; prepared using random oligonucleotide priming of the whole HBV genome). After 16 h of hybridization, the membranes were washed three times (20 min for each time) with 0.2 × SSC and 0.1% SDS at 52°C and then exposed to X-ray film for 16 h at −80°C.

### Quantitative RT-PCR

The Universal Probe Library was used to quantify C/EBPα, The expression level of a housekeeping gene, beta-2-microglobulin (B2M), was used as a reference gene. The relevant probes were selected from the Universal Probe Library Set (human), and the corresponding primers were combined with them. The primer pairs used in these experiments were:

CEBPα-forward (5′-CAACACTTGTATCTGGCCTCTG-3′),

CEBPα-reverse (5′-CGAGCAAAACCAAAACAAAAC-3′), and probe #03.

B2M-forward (5′-TTCTGGCCTGGAGGCTATC-3′).

B2M-reverse (5′-TCAGGAAATTTGACTTTCCATTC-3′), and probe #42.

The quantitative RT-PCR was performed using a Roche Light Cycler 480.

### RNA interference

The shRNAs used against CDKN1A (A1 and G1) and C/EBPα (05 and 06) were purchased from the National RNAi Core Facility, Taiwan and these were used to knock down endogenous expression of p21 and C/EBPα. Virus packaging was performed according to standard protocols as recommended by the National RNAi Core Facility, Taiwan. 1.3.ES2 cells were infected with lentivirus in 8 μg/ml polybrene and centrifuged at 1,200 × g for 20 minutes (multiplicity of infection = 3). After incubation for 24 hours, lentivirus-infected 1.3.ES2 cells were selected with 2 μg/ml of puromycin for three days and then used for the experiments.

The target sequences were:

pLKO.1-shCDKN1A-A1: CGCTCTACATCTTCTGCCTTA.

pLKO.1-shCDKN1A-G1: AGAGGTTCCTAAGAGTGCTGG.

pLKO.1-shC/EBPα: GCTGGAGCTGACCAGTGACAA.

### Co-immunoprecipitation assay

HepG2 cells were co-transfected with plasmids expressing C/EBPα and p21. Cell extracts were collected three days post-transfection. Cell lysate was co-incubated overnight at 4°C with 6 μg of rabbit polyclonal anti-p21 antibody (Genetex) or Protein G Mag Sepharose (GE). After the beads were washed, the protein complexes were eluted by heating in SDS sample buffer, which was followed by processing for immunoblot analysis.

### Chromatin immunoprecipitation (ChIP) assay

Rabbit polyclonal antibodies against C/EBPα (SC-61), p21 (sc-756) and rabbit IgG were obtained from Santa Cruz. The MAGnify chromatin immunoprecipitation system (Invitrogen) was used for the ChIP analysis, and was carried out as described in the manufacturer’s protocol. Briefly, the antibodies were coupled to Dynabeads Protein G at 4°C. Next, 5×10^6^ 1.3.ES2 cells were trypsinized and fixed with 1% formaldehyde, which was followed by lysis using lysis buffer containing protease inhibitor. DNA in the cross-linked chromatin preparation was fragmented by sonication to give a size range of 200 bp to 1kb. Immunoprecipitation was performed at 4°C and during this process the sheared chromatin was incubated with antibody-coated Dynabeads. After sequential washing with IP buffer l and 2, the formaldehyde crosslinking of the chromatin was reversed using reverse crosslinking buffer containing proteinase K at 55°C. The immunoprecipitated DNA was recovered from the supernatant using the DNA purification magnetic beads provided in the kit. This DNA was then subjected to quantitative RT-PCR or PCR analysis. The primers used for this analysis covering the HBV EnI, core and S promoter regions and are listed below.

HBV-coreQPCR-F(5′-TTGGGGGAGGAGATTAGGTT-3′).

HBV-coreQPCR-R(5′-TGCGCAGACCAATTTATGC-3′).

HBV-EnIQPCR-F(5′-GCATGCGTGGAACCTTTT-3′).

HBV-EnIQPCR-R (5′-CAAAACAAGCGGCTAGGAGT-3′).

HBV-1-F (5′-ATGTATTCAATCTAAGCAGGCT-3′).

HBV-197-R (5′-TCGGCAGAGGAGCCGAAAAG-3′).

HBV-544-F (5′-GAGACCACCGTGAACGCCCA-3′).

HBV-782-R (5′-ACAAGAGATGATCAGGCAGA-3′).

HBV-Sp-F (5′-GTGGTGGACTTCTCTCAATTTTC-3′).

HBV-Sp-R (5′-CGGTAAAAAAGGGACTCAAAGAT-3′).

### Luciferase assay

To analyze the effect of p21 on the activities of the HBV core promoters, HepG2 cells were co-transfected with pShIE/p21 or a mock control (pSh/IE) plasmid together with a reporter construct (pGL3/EnI+CP) and a SV40 promoter driven beta-galactosidase plasmid to monitor the transfection efficiency; the ratio of the three DNAs that formed the two experimental groups consisting was 4.5:4.5:1 in all cases. The transfection reactions were conducted in a 24-well plate with 3 × 10^5^ cells per well. Three days after cotransfection, the cells were collected and quantified using a Bright Glo luciferase assay kit (Promega Corporation, Madison, WI) and a Beta Glo luciferase assay kit (Promega Corporation, Madison, WI) to determine the luciferase and betagalactosidase activities, respectively. The luciferase signals were determined using a Hidex Chameleon luminescence reader (Hidex, Turku, Finland).

### Data analysis

Densitometric measurements of band intensities were made in order to quantify the proteins/nucleic acids and this was done with an AlphaEaseFC Imaging System software version 6.0.0 (Alpha Innotech Corp.). In all cases, the samples collected at the first time point were deemed to be 100%. All statistical analyses were performed using the 2-sided unpaired Student’s *t* test.

## Results

### Doxorubicin activates HBV replication by inducing p21 (Waf1/Cip1) expression

**Fig 1:**
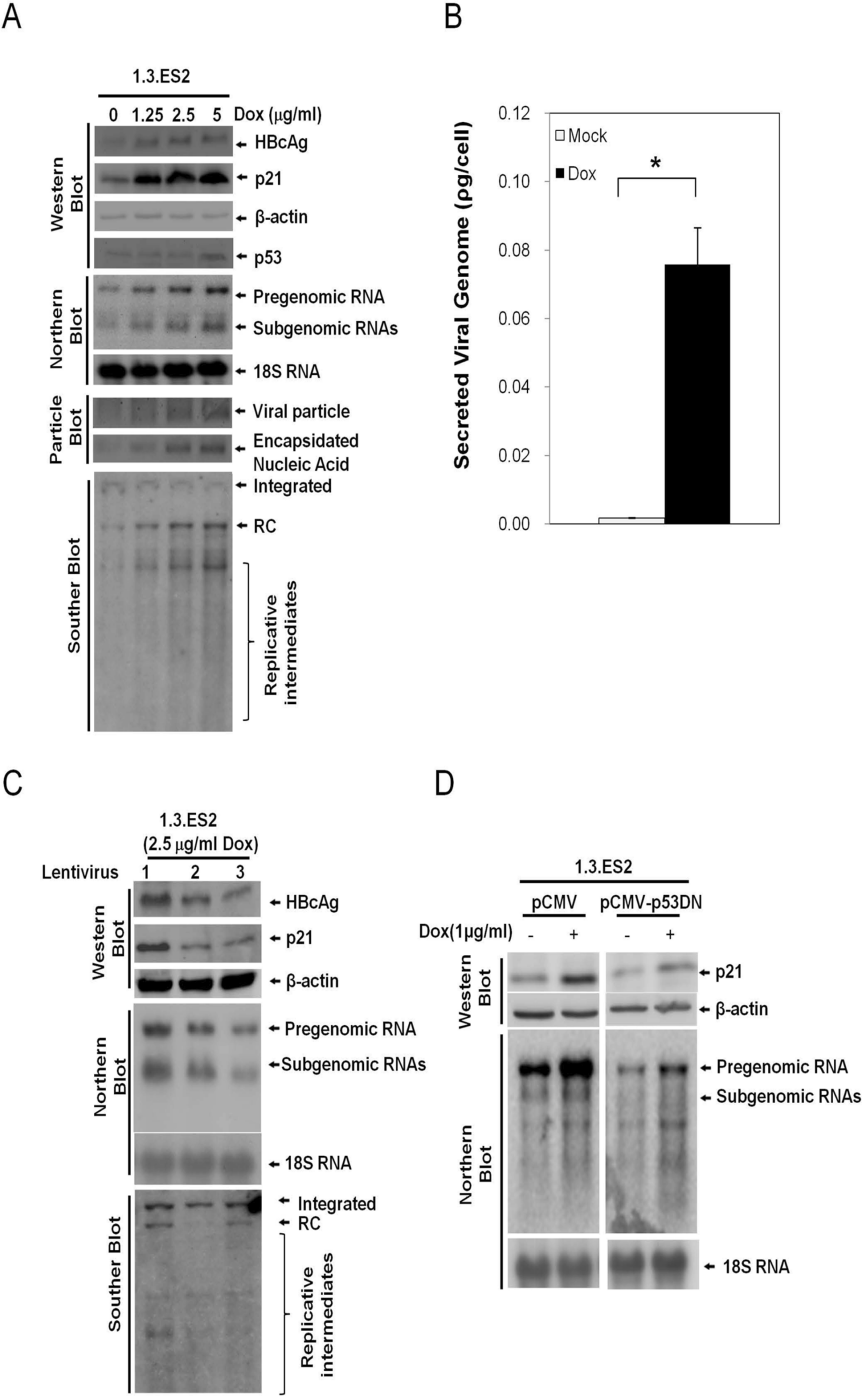
Doxorubicin modulates the expression levels of HBV, p21, and p53 in 1.3.ES2 cells. (A) The effect of doxorubicin treatment on HBV replication. 1.3.ES2 cells were treated with different doses of doxorubicin for 1 hour, and then their culture medium was replaced with fresh culture medium. Total DNA, total RNA and cell lysates were obtained three days after doxorubicin treatment for 1 hour for Southern blot, Northern blot and particle blot/Western blot analyses, respectively. The expression levels of HBV transcripts, viral particles, HBcAg, p21, and p53 are shown. (B) The effect of doxorubicin on the modulation of viral particle generation. 1.3.ES2 cells were treated with 5 μg/ml doxorubicin for 1 hour then their culture medium was replaced with fresh culture medium for three days. Secreted viral particles were then obtained from the culture medium and analyzed to measure their content in terms of viral genomes by quantitative RT-PCR assay. (C) P21 participates in doxorubicin active HBV replication. 1.3.ES2 cells were infected with lentivirus as indicated for 24 hours, which was followed by doxorubicin treatment for 1 hour. The expression levels of total DNA, HBcAg, p21, and HBV transcripts were then analyzed by Western blot and Northern blot assays three days after doxorubicin treatment. Lane 1, control lentivirus expressing shLuc RNA. Lanes 2 and 3 are lentiviruses carrying shRNAs against p21 (sh-CDKN1A-A1 and sh-CDKN1A-G1, respectively). (D) The role of p53 in doxorubicin-mediated HBV activation. 1.3.ES2 cells were transfected with plasmid expressing a domain negative form of p53 (pCMV-p53DN) or mock vector (pCMV) and these two experimental groups were then subjected to doxorubicin treatment for 1 hour. The cell lysates were harvested three days after doxorubicin treatment. The expression levels of p21 protein and HBV transcripts were determined by Western blot and Northern blot analysis, respectively.

To examine the effect of doxorubicin on HBV activation, HBV-producing HepG2 cells (1.3.ES2 cells) were treated with different doses of doxorubicin and then harvested to analyze their HBV replication capability. Southern blot, Northern blot and particle blot analyses showed that doxorubicin significantly enhanced HBV replication in a dose-dependent manner as is evident by the increased levels of HBV nucleic acid, HBV transcripts and viral nucleocapsids, respectively (Fig 1A, fifth and last three panels). Dose-dependent increases in intracellular HBV core protein (HBcAg) and secreted HBV viral particles (secreted viral genome in culture medium) were also detected by Western blot assay and quantitative RT-PCR analysis, respectively (Fig 1A, the first panel from top and Fig 1B). Similar results in terms of HBV activation by doxorubicin has been reported previously using a different HBV-producing hepatoma cell line, 2.2.15 cells [12].

We found that doxorubicin treatment significantly decreased the proliferation rate of 1.3.ES2 cells (data not shown). As a consequence, we examined the expression level of the cell cycle regulator p21 in response to doxorubicin treatment. As expected, the expression level of p21 was increased by doxorubicin in a dose-dependent manner (Fig 1A, second panel). To further confirm the role of p21 in doxorubicin-mediated HBV activation, we knocked down p21 transcripts in the doxorubicin-treated 1.3.ES2 cells and then analyzed the replicative capacity of HBV. Interestingly, knock-down of p21 completely abolished the activation of HBV by doxorubicin as revealed by the reductions in HBV replicative genomes, core proteins and viral transcripts (Fig 1C). Moreover, overexpression of a dominant-negative p53 was shown to partially block the elevation in p21 transcription associated with increased HBV replication during doxorubicin treatment (Fig 1D). These results indicate that doxorubicin upregulates the expression levels of p53 and p21 in 1.3.ES2 cells and this subsequently activates HBV replication.

### Overexpression of p21 (Waf1/Cip1) is able to activate HBV expression

**Fig 2:**
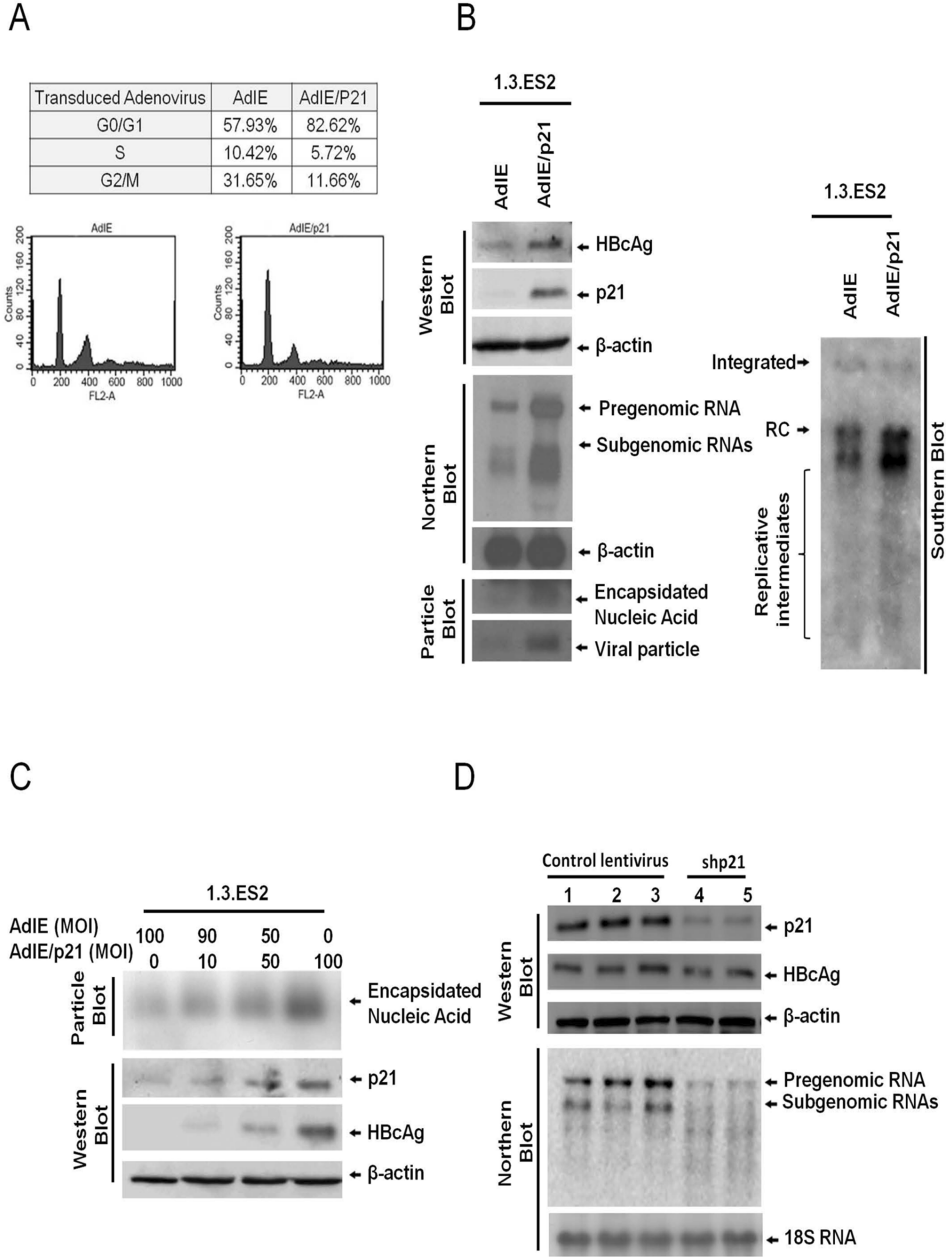
Expression level of p21 modulates HBV replication in 1.3.ES2 cells. (A) Cell cycle analysis of 1.3.ES2 cells with p21 overexpression. 1.3.ES2 cells were transduced with AdIE (a mock control adenovirus) or with AdIE/p21 (an adenovirus carrying the p21 gene). Next the cell cycle distribution was analyzed by flow cytometry assay. (B) The effect of p21 overexpression on the modulation of HBV replication. Cell lysates were extracted from adenoviruses-infected 1.3.ES2 cells, and the levels of HBV genomes and HBV transcripts were examined by Southern and Northern blot analysis using a HBV-specific probe. The expression of beta-actin was used as the loading control in the Northern blot. The expression of HBV nucleocapsids and the embedded viral genome were detected by particle blot analysis. The expression levels of HBcAg and p21 were analyzed by Western blot assay. (C) The dosage effect of p21 on the modulation of HBV replication. 1.3.ES2 cells were transduced with different amounts of AdIE and/or AdIE/p21, and equal amounts of cell lysates were subjected to native agarose gel electrophoresis to allow particle blot analysis. The encapsidated viral genomes were detected using a HBV-specific probe. (D) Effect of p21 knock-down on the modulation of HBV replication. 1.3ES2 cells were infected with lentiviruses that expressed control shRNA or shRNA against p21. Four days after lentivirus infection, the cells were harvested for Northern blot and Western blot analysis. Lanes 1, 2 and 3 contained control virus samples (vector alone, shRFP and shLuc, respectively), while Lanes 4 and 5 contained cells transfected with two different shCDKN1As; these were sh-CDKN1A-A1 and sh-CDKN1A-G1, respectively.

Previous studies have shown that HBV replication is closely associated with the cell cycle and is higher during the G0/G1 phase [15, 16]. To clarify the role of p21 in the modulation of HBV replication, 1.3.ES2 cells were transduced with adenovirus carrying p21 gene and the replicative capacity of HBV in response to p21 overexpression was analyzed (Fig 2). The percentage of cells in G0/G1 phase was significantly increased from 57.93% to 82.62% by the overexpression of p21 (Fig 2A). At the same time, the expression levels of HBV genomes, HBcAg, HBV transcripts, and viral nuclecapsids were dramatically enhanced by p21 overexpression (Fig 2B). The levels of intracellular HBcAg and encapsidated viral nucleic acids were positively correlated with the level of p21 protein expression (Fig 2C). Furthermore, knock-down of p21 expression in 1.3.ES2 cells was found to markedly attenuate the expression of HBV transcripts and HBcAg (Fig 2D). These results showed that modulation of p21 level alone is capable of regulating HBV transcription and viral nucleocapsid formation, suggesting that p21 is a critical regulator during HBV replication.

### Investigation of the p21 (Waf1/Cip1) responsive elements embedded within the HBV genome

**Fig 3:**
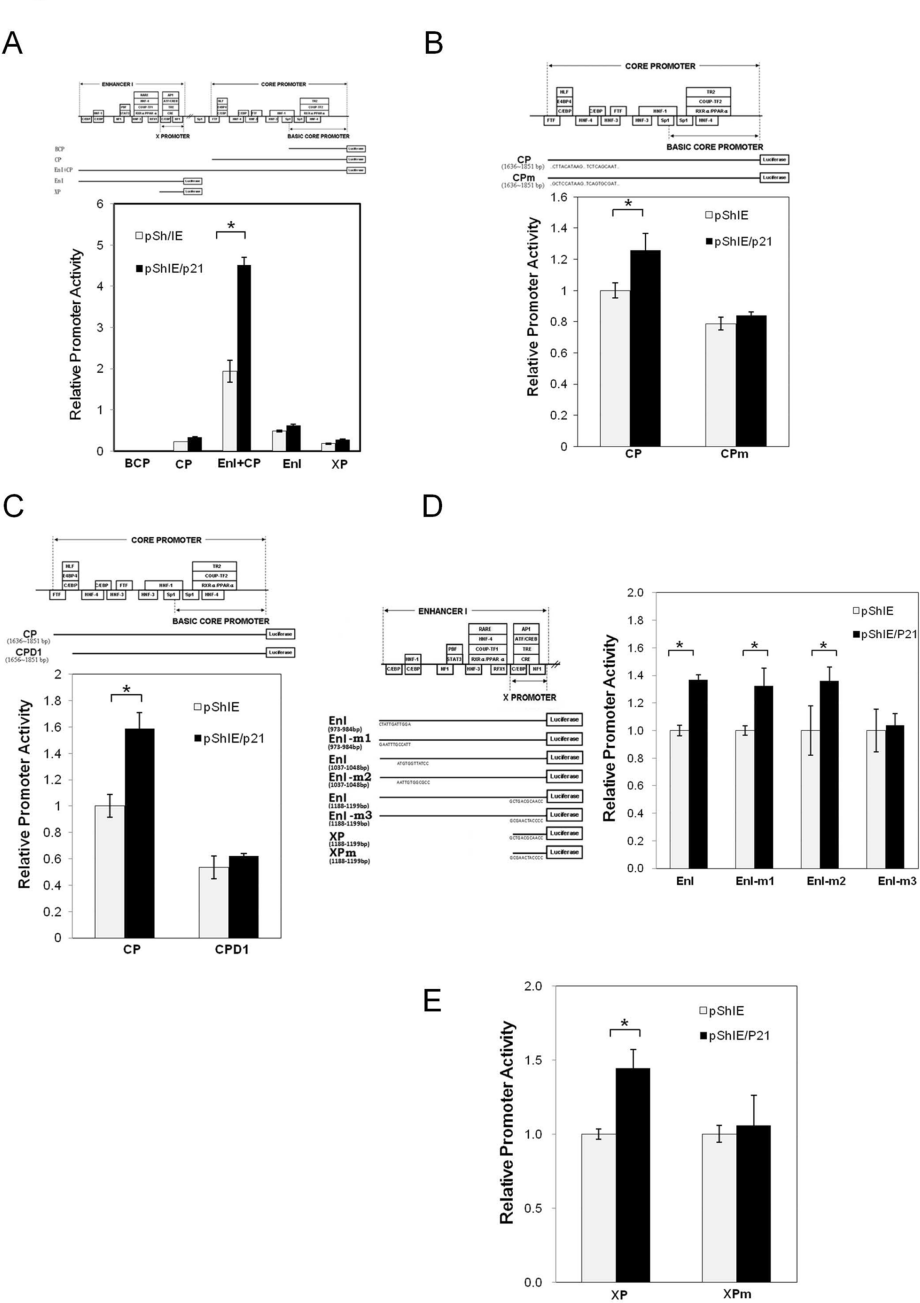
Identification of p21 responsive elements within the HBV genome. HepG2 cells were co-transfected with pShIE/p21 or mock control (pShIE) together with an appropriate indicated luciferase reporter plasmid. These were (A) luciferase reporter plasmids driven by the various HBV promoters, namely the BCP (basic core promoter), the CP (core promoter), EnI+CP (enhancer I plus the core promoter), EnI (enhancer I) and XP (the X promoter), (B) luciferase reporter plasmid driven by the HBV core promoter (CP) and the HBV core promoter with a deletion (CPD1), or the HBV core promoter with a mutation at two C/EBP binding sites (CPm), (D) luciferase reporter plasmid driven by the HBV EnI and EnI with mutations at three different C/EBP binding sites (EnI-m1, EnI-m2 and EnI-m3). (E) luciferase reporter plasmid driven by the HBV X promoter and HBV X promoter with a mutation at the C/EBP binding sites (XPm). Cells were co-transfected with pSV40-beta-galactosidase plasmid in order that the galactosidase activity of each sample could be used for normalization. After transfection for 3 days, the cell lysates were extracted to examine the luciferase and galactosidase activity levels of the samples. The schematic shows the relative location of the promoter sequences used in the various assays. The relative luciferase activity of the different promoters using cells with or without p21 overexpression are shown in the bar chart. The grey bar represents the cells without p21 overexpression, and the black bar represents the cells with p21 overexpression. The results are shown as the relative firefly luciferase activity levels normalized against each sample's beta-galactosidase activity; the experiments were carried out in triplicate. The relative ratios of the luciferase activity of the cells with or without p21 overexpression are also shown below the chart. *, *p* < 0.01, 2-sided unpaired t test.

In order to explore the mechanism underlying HBV activation by p21 overexpression, a series of reporter assays were carried out to identify the responsive element involved in the p21-mediated activation of HBV pgRNA transcription (Fig 3). By cotransfecting HepG2 cells with a p21-expressing plasmid and different reporters containing different viral promoter regions, namely the basic core promoter (BCP), the core promoter (CP), enhancer I (EnI), the HBx promoter (XP) and EnI plus CP, we found that the overexpression of p21 significantly induced the promoter activity levels of the CP, EnI, XP, and EnI plus CP. Of these constructs, namely the reporters-containing CP, EnI and XP, showed only a slight inductions (1.3–1.5 fold) of promoter activity by p21 overexpression. However, EnI plus CP showed a synergistic effect on the induction of promoter activity (2.3 fold). BCP alone did not respond to p21 overexpression (Fig 3A). To further clarify the molecule mechanism by which p21 up-regulates pgRNA synthesis, reporters with deletion(s) or mutation(s) in the CP, EnI and XP regions were used to map the p21-responsive element(s) (Fig 3B–3E). We found that overexpression of p21 failed to induce the promoter activity of CPD1, a reporter with a 20 bp deletion at the N-terminus of the CP [23], indicating that the p21 responsive element is likely to be located within a region (nucleotides 1636–1656) of the core promoter (Fig 3B). A survey of the known regulatory elements embedded within the region (nucleotides 1636–1656), suggested that a C/EBP binding element might be the responsive element required for p21-mediated CP activation (Fig 3B). A previous study identified five C/EBP binding elements that are located separately within the CP, EnI and XP regions [26, 27]. Interestingly, all these promoters were identified to be activated by p21 overexpression (Fig 3A). To further investigate the requirement for the C/EBPα binding element in p21-mediated CP activation, point mutations within the C/EBPα binding elements (CPm) of the CP reporter were generated. These reporter assays revealed that the overexpression of p21 significantly enhanced the promoter activity of the intact CP, but failed to enhance the promoter activity of Cpm in which the C/EBPα binding sites were mutated (Fig 3C). Interestingly, a C/EBPα-binding site mutation at nucleotides 1188–1199 (EnI-m3 in Fig 3D or Xpm in Fig 3E) significantly abolished the activation of the corresponding promoter activity (EnI or XP) upon p21 overexpression. Whereas, mutations at the C/EBPα-binding sequence 973–984 or 1037–1048 (EnI-m1 or EnI-m2) did not affect the activation of EnI promoter activity by p21. These findings suggest that the p21 responsive elements that are embedded within the HBV genome are very likely to be associated with the C/EBP binding sites at 1636–1851 and 1188–1199.

### Elevation of C/EBPα expression by p21 (Waf1/Cip1) in doxorubicin-treated hepatocytes

**Fig 4:**
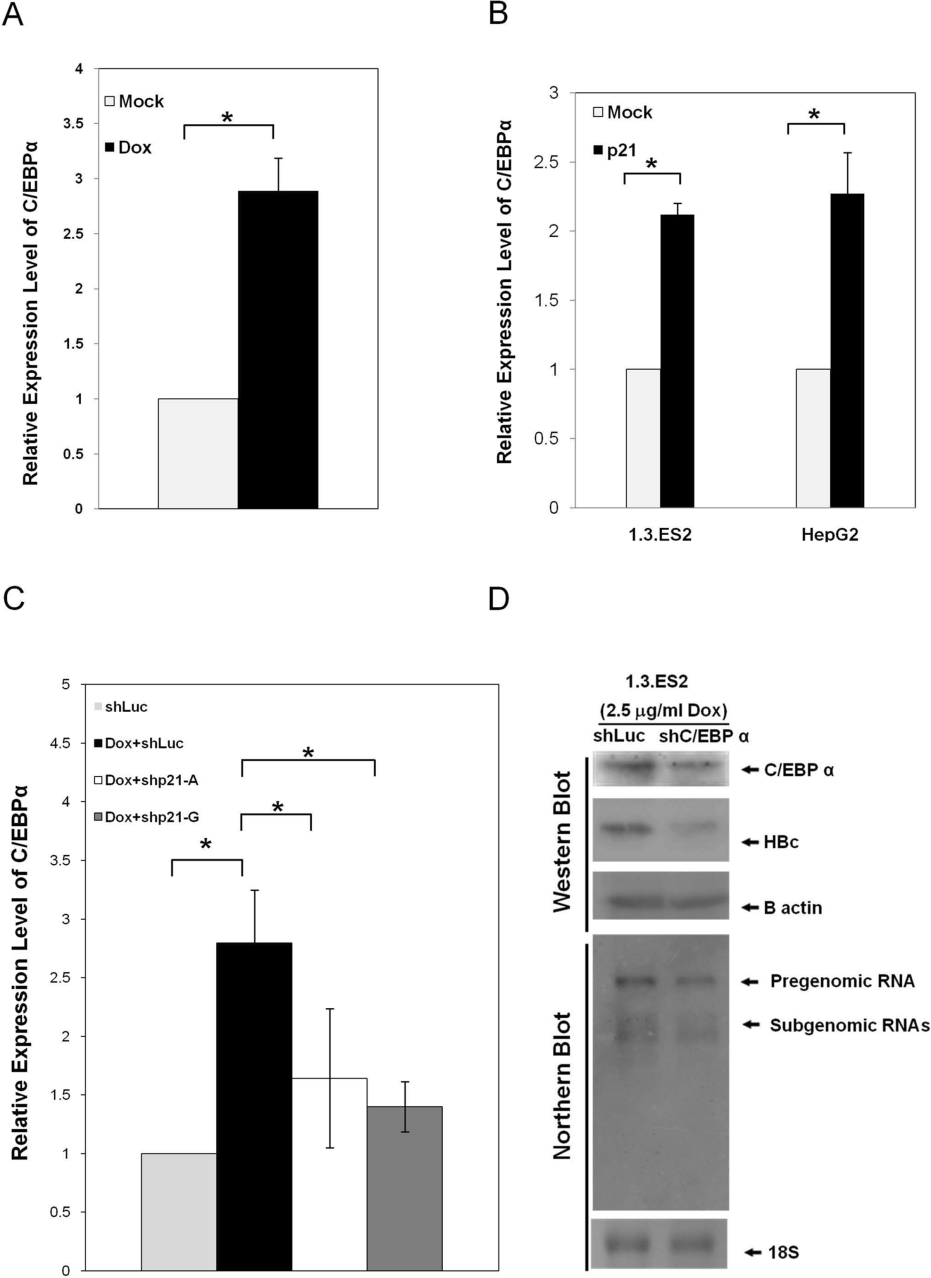
The elevation of C/EBPα by doxorubicin and its impact on activation of HBV replication. (A) The elevation of C/EBPα by doxorubicin. 1.3.ES2 was treated with doxorubicin for 1 hour and then the culture medium was replaced with fresh culture medium for three days. Next, total RNA was harvested in order to carry out quantitative RT-PCR. (B) The elevation of C/EBPα by p21 over-expression. 1.3.ES2 cells or HepG2 cells over-expressing p21 were harvested for analysis of the expression level of C/EBPα by quantitative RT-PCR. (C) The role of p21 in doxorubicin-mediated C/EBPα elevation. 1.3.ES2 cells were infected with lentivirus expressing p21 shRNA. Twenty-four hours post-infection, cells were treated with doxorubicin for 1 hour, which was followed by replacing the culture medium with fresh culture medium. Culturing was then continued for 3 days. Next, total RNA was harvested for analysis of the expression level of C/EBPα by quantitative RT-PCR. The expression level of B2M was used as a reference control. (D) 1.3.ES2 cells were infected with lentivirus expressing C/EBPα shRNA for 24 hours, treated with doxorubicin for 1 hour, and this was followed by culturing in fresh culture medium for 3 days. The expression levels of HBV transcripts, HBcAg and C/EBPα were analyzed by Northern blot and Western blot.analysis, *, *P* < 0.01, 2-sided unpaired t test.

The expression levels of C/EBPα mRNA in response to doxorubicin treatment were analyzed by quantitative RT-PCR assay. 1.3.ES2 cells that had been treated with doxorubicin showed a significant elevation in the expression levels of C/EBPα (Fig 4A). Interestingly, over-expression of p21 in HepG2 or 1.3.ES2 cells was sufficient to up-regulate the level of C/EBPα (Fig 4B). To further investigate the role of p21 in the doxorubicin-mediated elevation of C/EBPα, p21 knock-down 1.3.ES2 cells were treated with doxorubicin and harvested for analysis of C/EBPα expression. Quantitative RT-PCR analysis showed that knock-down of p21 expression significantly lowered the amount of C/EBPα induction brought about by doxorubicin treatment (Fig 4C). Moreover, down-regulation of C/EBPα expression by RNAi resulted in a coincident decrease in HBV replicative activity in the doxorubicin-treated 1.3.ES2 cells (Fig 4D). These findings suggest that doxorubicin treatment is able to up-regulate C/EBPα expression, very likely via the activation of p21, and that these elevated levels of C/EBPα may play an important role in doxorubicin-mediated activation of HBV replication.

### *In vivo* binding of C/EBPα and its interaction with p21 (Waf1/Cip1) on the HBV promoter(s)

**Fig 5:**
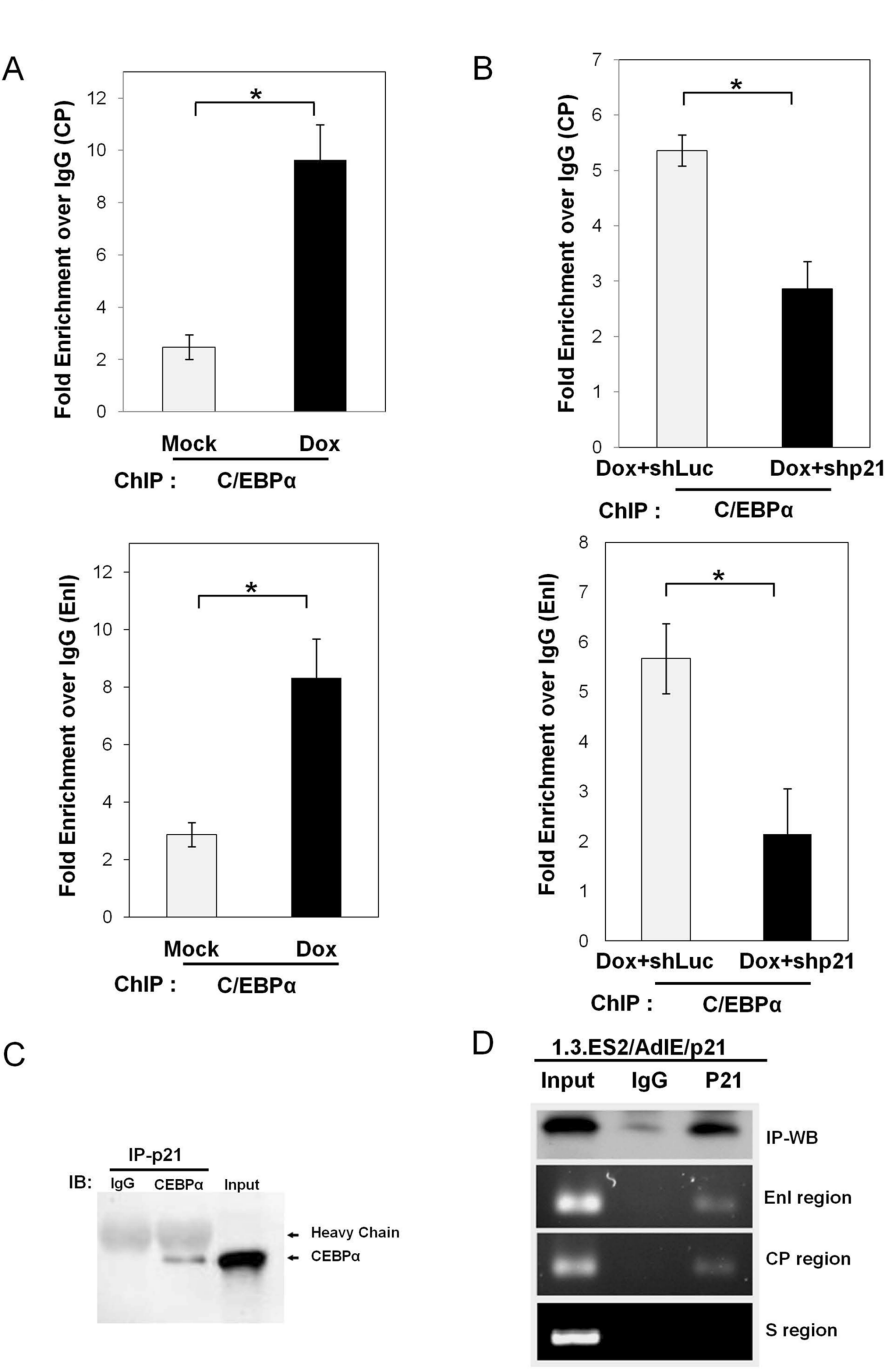
Binding of C/EBPα to the HBV promoter(s) is modulated by doxorubicin and p21. (A) Doxorubicin enhances C/EBPα recruitment to CP and EnI. 1.3.ES2 cells and doxorubicin-treated 1.3.ES2 cells were harvested in order to carry out ChIP assays. The nuclear extracts were co-incubated with IgG or anti-C/EBPα antibody and the content of the enriched chromatin fragments obtained were analyzed by quantitative RT-PCR. (B) P21 modulates C/EBPα recruitment to CP and EnI in doxorubicin-treated 1.3.ES2 cells. 1.3.ES2 cells were infected with lentivirus expressing p21 shRNA or control shRNA (shLuc) and this was followed by doxorubicin treatment. The nuclear extracts were harvested for ChIP analysis of the binding efficiency of C/EBPα to CP and EnI. The enrichments of chromatin fragments were determined by quantitative RT-PCR. (C) The protein-protein interaction between p21 and C/EBPα. HepG2 cells were transfected with plasmids expressing C/EBPα and p21 and then the cell lysates were precipitated with anti-p21 antibody, followed by immunoblotting with IgG or anti-CEBPα antibody. The co-immunoprecipitation assay shows the interaction between C/EBPα and p21. (D) A ChIP assay was used to determine the *in vivo* binding of p21 onto the HBV promoter region in 1.3.ES2 cells. The 1.3.ES2 cells were transduced with AdIE/p21 for 3 days and then collected for the ChIP assay using IgG and p21 antibody. Three pairs of PCR primers for the detection of the EnI region, CP region and S region were designed and used to amplify the immunoprecipitated HBV DNA fragments. *, *p* < 0.01, 2-sided unpaired t test.

To assess whether doxorubicin increases the binding of C/EBPα to its responsive element within the HBV promoter(s) *in vivo*, nuclear extracts from doxorubicin-treated 1.3.ES2 cells were harvested for a chromatin immunoprecipitation (ChIP) assay (Fig 5A). As seen in Fig 5A, doxorubicin treatment significantly enhanced the recruitment of C/EBPα onto the core promoter and EnI. On the other hand, knock-down of p21 in the doxorubicin-treated 1.3.ES2 cells dramatically reduced the recruitment of C/EBPα onto the core promoter and EnI (Fig 5B). These findings suggest that p21 plays an important role in doxorubicin-mediated C/EBPα recruitment onto the HBV core promoter and EnI. Previous reports have shown that p21 is able to bind to transcription factors and regulate their functions [19, 20, 28]. C/EBPα is a hepatocyte-enriched transcription factor (HETF) that is able to interact with p21 [20]. Indeed, our co-immunoprecipitation assay showed that C/EBPα and p21 were able to form a protein complex in hepatoma cells (Fig 5C). This suggests the possibility that p21 may indirectly bind to HBV promoters through its interaction with C/EBPα, and that the p21-C/EBPα complex may stabilize this binding and this, in turn, exert a regulatory effect on HBV activation. To assess whether p21 binds to the HBV promoters, chromatin immunoprecipitation (ChIP) assay was performed using AdIE/p21-transduced 1.3.ES2 cells. The ChIP assay showed that p21 was able to bind to the CP and EnI regions, but not to a region corresponding to the HBV surface region (Fig 5D). It should be noted that both the CP and EnI regions contain C/EBP binding elements and these have been shown earlier to be activated by p21 overexpression using a reporter assay (Fig 3). These findings raise the possibility of p21 cooperating with C/EBPα in order to bind to the C/EBP binding elements within the HBV promoters.

## Discussion

Several clinical studies have reported that chemotherapy, including doxorubicin treatment, reactivates HBV replication in cancer patients, but the detailed mechanism by which HBV is reactivated has not yet been identified. It has also been noted that chemotherapeutic agents are able to induce p21 expression and this can bring about cell cycle G0/G1 arrest [29, 30]. In the current study, we showed that doxorubicin treatment activated HBV replication in an HBV-producing cell line and this was associated with concurrent increases in the levels of p53 and p21 (Fig 1A). Expression of dominant-negative p53 was found to attenuate the elevation of p21 induced by doxorubicin treatment (Fig 1D), indicating that p53 activates the expression of p21 in response to the cell stress induced by doxorubicin. Further study showed that over-expression of p21 was sufficient to enhance HBV replication (Fig 2B), whereas knock-down of p21 significantly decreased the expression level of HBV (Fig 2D). Reporter assays demonstrated that the HBV promoters CP, EnI, EnI plus CP, and XP were activated by p21 expression (Fig 3A). Deletion and mutation analyses in the HBV CP, EnI and XP propmoter regions revealed that the C/EBP binding elements (nucleotides 1636–1851 and 1188–1199), which are located within the HBV CP and EnI region, (as well as in XP), are responsive to p21-mediated activation (Fig 3B–3E). In addition, we showed that the expression of p21 not only increased the expression level of C/EBPα, but also played an important role in the recruitment of C/EBPα to its responsive element (Figs 4B and 5B). Our study revealed the *in vivo* binding of p21 to the C/EBP responsive element of the HBV promoters CP and EnI, probably occurs via the formation of a p21-C/EBPα complex (Fig 5C and 5D). Taken together, these findings suggest that doxorubicin evokes the expression of p21 which then elevates the expression level of C/EBPα and this complex then brings about the activation of HBV replication by interacting with HBV promoters.

Several transcription factors bound to the HBV core promoter are known to be responsible for the transcription of HBV pregenomic RNA (pgRNA), which plays a pivotal role in the HBV life cycle [26]. C/EBPα, a hepatocyte-enriched transcription factor, has been suggested to be involved in interactions with five responsive sites on the HBV promoters, including EnI, CP and XP [26, 27, 31]. Enhancer II located within the HBV core promoter has also been reported to respond to the expression of C/EBPα [31]. IL-4 treatment has been shown to decrease the amount of C/EBPα and significantly suppressed HBV core promoter activity [32]. Disruption of the interaction between C/EBPα and EnI by *Phyllanthus amarus* also is known to inhibit HBV RNA transcription [33]. These findings demonstrate that the level of C/EBPα plays an important role in modulating HBV replication. In the current study, we demonstrated that C/EBPα is a critical mediator of doxorubicin-mediated HBV activation, as is evident from the following: (i) the expression level of C/EBPα is modulated by doxorubicin and p21 exerts an effect on this (Fig 4A and 4B); (ii) the presence of p21 is crucial to C/EBPα recruitment in doxorubicin-treated cells (Fig 5A and 5B); and (iii) p21 and C/EBPα would seem to form a complex that interacts with various HBV promoters (Fig 5C and 5D, and see the discussion below). Since p21 itself is not a DNA binding protein, the loading of p21 onto various HBV promoters is likely to be through its interaction with C/EBPα. Since the formation of a p21-C/EBPα complex has been reported to stabilize p21 protein level by blocking the proteolytic degradation of p21 [34], the stabilized p21-C/EBPα complex is likely to contribute to maintaining the activated status of HBV replication. However, the biological function of p21, when it is loaded onto the HBV promoters, remains to be elucidated.

HBV replication has been shown to be stimulated by the expression of HBx [35, 36]. We have also showed that HBx expression is essential for optimal transcription of viral transcripts from covalently closed circular DNA (cccDNA) [21]. Interestingly, several studies have linked HBx to an increase in the expression level of p21 in hepatocytes [37, 38]. HBx has been reported to elevate p21 expression by targeting the HBx responsive element embedded within the p21 promoter [37]. HBx has also been reported to interact with C/EBPα, which strongly activates HBV core promoter activity as well as the pgRNA transcription in a synergistic manner [39]. This raises the possibility that p21, C/EBPα and HBx might form a functional complex and this complex is then able to cooperate in order to enhance HBV replication by binding to the HBV promoters. Taken together, we suggest that doxorubicin induces an increase in p21 expression in hepatocyte cells when it is used as an anti-cancer drug. This elevation in the level of p21 up-regulates the expression level of C/EBPα and this consequently enhances its recruitment to CP and EnI, which then in turn enhances HBV transcription and thus viral replication. In summation, in this study we outline and provide evidence for a possible mechanism that underlies HBV reactivation during doxorubicin chemotherapy.

## References

[1] Glebe D. Recent advances in hepatitis B virus research: a German point of view. World J Gastroenterol. 2007;13(1):8–13. . 17206750 10.3748/wjg.v13.i1.8 PMC4065879

[2] Kao JH, Chen DS. Global control of hepatitis B virus infection. Lancet Infect Dis. 2002;2(7):395–403. Epub 2002/07/20. doi: S1473309902003158 [pii]. . 12127351 10.1016/s1473-3099(02)00315-8

[3] Carey I, Harrison PM. Monotherapy versus combination therapy for the treatment of chronic hepatitis B. Expert opinion on investigational drugs. 2009;18(11):1655–66. Epub 2009/10/27. 10.1517/13543780903241599 . 19852566

[4] de Jongh FE, Janssen HL, de Man RA, Hop WC, Schalm SW, van Blankenstein M. Survival and prognostic indicators in hepatitis B surface antigen-positive cirrhosis of the liver. Gastroenterology. 1992;103(5):1630–5. . 1426884 10.1016/0016-5085(92)91188-a

[5] Fattovich G, Brollo L, Giustina G, Noventa F, Pontisso P, Alberti A, et al Natural history and prognostic factors for chronic hepatitis type B. Gut. 1991;32(3):294–8. Epub 1991/03/01. 2013423 10.1136/gut.32.3.294 PMC1378837

[6] Mast EE, Alter MJ, Margolis HS. Strategies to prevent and control hepatitis B and C virus infections: a global perspective. Vaccine. 1999;17(13–14):1730–3. Epub 1999/04/09. . 10194830 10.1016/s0264-410x(98)00415-0

[7] McQuillan GM, Coleman PJ, Kruszon-Moran D, Moyer LA, Lambert SB, Margolis HS. Prevalence of hepatitis B virus infection in the United States: the National Health and Nutrition Examination Surveys, 1976 through 1994. American journal of public health. 1999;89(1):14–8. Epub 1999/02/13. 9987458 10.2105/ajph.89.1.14 PMC1508496

[8] Ide Y, Ito Y, Takahashi S, Tokudome N, Kobayashi K, Sugihara T, et al Hepatitis B virus reactivation in adjuvant chemotherapy for breast cancer. Breast Cancer. Epub 2010/07/27. 10.1007/s12282-010-0213-x . 20658270

[9] Koo YX, Tay M, Teh YE, Teng D, Tan DS, Tan IB, et al Risk of hepatitis B virus (HBV) reactivation in hepatitis B surface antigen negative/hepatitis B core antibody positive patients receiving rituximab-containing combination chemotherapy without routine antiviral prophylaxis. Ann Hematol. 2011;90(10):1219–23. Epub 2011/04/27. 10.1007/s00277-011-1241-0 . 21520001

[10] Yeo W, Chan PK, Zhong S, Ho WM, Steinberg JL, Tam JS, et al Frequency of hepatitis B virus reactivation in cancer patients undergoing cytotoxic chemotherapy: a prospective study of 626 patients with identification of risk factors. Journal of medical virology. 2000;62(3):299–307. . 11055239 10.1002/1096-9071(200011)62:3<299::aid-jmv1>3.0.co;2-0

[11] Yeo W, Lam KC, Zee B, Chan PS, Mo FK, Ho WM, et al Hepatitis B reactivation in patients with hepatocellular carcinoma undergoing systemic chemotherapy. Annals of oncology: official journal of the European Society for Medical Oncology / ESMO. 2004;15(11):1661–6. 10.1093/annonc/mdh430 . 15520068

[12] Hsu CH, Hsu HC, Chen HL, Gao M, Yeh PY, Chen PJ, et al Doxorubicin activates hepatitis B virus (HBV) replication in HBV-harboring hepatoblastoma cells. A possible novel mechanism of HBV reactivation in HBV carriers receiving systemic chemotherapy. Anticancer research. 2004;24(5A):3035–40. . 15517913

[13] He G, Siddik ZH, Huang Z, Wang R, Koomen J, Kobayashi R, et al Induction of p21 by p53 following DNA damage inhibits both Cdk4 and Cdk2 activities. Oncogene. 2005;24(18):2929–43. Epub 2005/03/01. 1208474 [pii] 10.1038/sj.onc.1208474 . 15735718

[14] Qin LF, Ng IO. Induction of apoptosis by cisplatin and its effect on cell cycle-related proteins and cell cycle changes in hepatoma cells. Cancer Lett. 2002;175(1):27–38. Epub 2001/12/06. doi: S0304383501007200 [pii]. . 11734333 10.1016/s0304-3835(01)00720-0

[15] Huang YQ, Wang LW, Yan SN, Gong ZJ. Effects of cell cycle on telomerase activity and on hepatitis B virus replication in HepG2 2.2.15 cells. Hepatobiliary & pancreatic diseases international: HBPD INT. 2004;3(4):543–7. . 15567742

[16] Ozer A, Khaoustov VI, Mearns M, Lewis DE, Genta RM, Darlington GJ, et al Effect of hepatocyte proliferation and cellular DNA synthesis on hepatitis B virus replication. Gastroenterology. 1996;110(5):1519–28. Epub 1996/05/01. doi: S0016508596002259 [pii]. . 8613059 10.1053/gast.1996.v110.pm8613059

[17] Chong CL, Chen ML, Wu YC, Tsai KN, Huang CC, Hu CP, et al Dynamics of HBV cccDNA expression and transcription in different cell growth phase. Journal of biomedical science. 2011;18:96 10.1186/1423-0127-18-96 22208719 PMC3262020

[18] SHIYANOV PAVEL B S, ADAMI GUY, KOKONTIS JOHN, HAY NISSIM, ARROYO MAY, RAYCHAUDHURI1 AMAP. p21 Disrupts the Interaction between cdk2 and the E2F-p130 Complex. Mol Cell Biol. 1996;16(3):737–44. 8622674 10.1128/mcb.16.3.737 PMC231053

[19] Kitaura H, Shinshi M, Uchikoshi Y, Ono T, Iguchi-Ariga SM, Ariga H. Reciprocal regulation via protein-protein interaction between c-Myc and p21(cip1/waf1/sdi1) in DNA replication and transcription. The Journal of biological chemistry. 2000;275(14):10477–83. Epub 2000/04/01. . 10744738 10.1074/jbc.275.14.10477

[20] Harris TE, Albrecht JH, Nakanishi M, Darlington GJ. CCAAT/enhancer-binding protein-alpha cooperates with p21 to inhibit cyclin-dependent kinase-2 activity and induces growth arrest independent of DNA binding. The Journal of biological chemistry. 2001;276(31):29200–9. Epub 2001/05/23. 10.1074/jbc.M011587200 M011587200 [pii]. . 11369759

[21] Chou YC, Jeng KS, Chen ML, Liu HH, Liu TL, Chen YL, et al Evaluation of transcriptional efficiency of hepatitis B virus covalently closed circular DNA by reverse transcription-PCR combined with the restriction enzyme digestion method. J Virol. 2005;79(3):1813–23. Epub 2005/01/15. 10.1128/JVI.79.3.1813-1823.2005 15650205 PMC544084

[22] Galibert F, Mandart E, Fitoussi F, Tiollais P, Charnay P. Nucleotide sequence of the hepatitis B virus genome (subtype ayw) cloned in E. coli. Nature. 1979;281(5733):646–50. Epub 1979/10/25. . 399327 10.1038/281646a0

[23] Hong MH, Chou YC, Wu YC, Tsai KN, Hu CP, Jeng KS, et al Transforming growth factor-beta1 suppresses hepatitis B virus replication by the reduction of hepatocyte nuclear factor-4alpha expression. PloS one. 2012;7(1):e30360 Epub 2012/01/26. 10.1371/journal.pone.0030360 22276183 PMC3262823

[24] Calvert J, Summers J. Two regions of an avian hepadnavirus RNA pregenome are required in cis for encapsidation. J Virol. 1994;68(4):2084–90. Epub 1994/04/01. 7511168 10.1128/jvi.68.4.2084-2090.1994 PMC236682

[25] Biermer M, Puro R, Schneider RJ. Tumor necrosis factor alpha inhibition of hepatitis B virus replication involves disruption of capsid Integrity through activation of NF-kappaB. J Virol. 2003;77(7):4033–42. Epub 2003/03/14. 12634363 10.1128/JVI.77.7.4033-4042.2003 PMC150632

[26] Moolla N, Kew M, Arbuthnot P. Regulatory elements of hepatitis B virus transcription. J Viral Hepat. 2002;9(5):323–31. Epub 2002/09/13. doi: 381 [pii]. . 12225325 10.1046/j.1365-2893.2002.00381.x

[27] Lopez-Cabrera M, Letovsky J, Hu KQ, Siddiqui A. Multiple liver-specific factors bind to the hepatitis B virus core/pregenomic promoter: trans-activation and repression by CCAAT/enhancer binding protein. Proceedings of the National Academy of Sciences of the United States of America. 1990;87(13):5069–73. 2367525 10.1073/pnas.87.13.5069 PMC54263

[28] Snowden AW, Anderson LA, Webster GA, Perkins ND. A novel transcriptional repression domain mediates p21(WAF1/CIP1) induction of p300 transactivation. Mol Cell Biol. 2000;20(8):2676–86. Epub 2000/03/25. 10733570 10.1128/mcb.20.8.2676-2686.2000 PMC85483

[29] Xiong J, Hu L, Li Y, Dou L, Cai P, Tang Z, et al Effect of survivin regulation of transcription level by p21waf1 overexpression in HepG2 hepatocellular carcinoma cells. Journal of Huazhong University of Science and Technology Medical sciences = Hua zhong ke ji da xue xue bao Yi xue Ying De wen ban = Huazhong keji daxue xuebao Yixue Yingdewen ban. 2008;28(3):308–13. 10.1007/s11596-008-0318-z . 18563330

[30] Yun C, Lee JH, Park H, Jin YM, Park S, Park K, et al Chemotherapeutic drug, adriamycin, restores the function of p53 protein in hepatitis B virus X (HBx) protein-expressing liver cells. Oncogene. 2000;19(45):5163–72. 10.1038/sj.onc.1203896 . 11064453

[31] Lopez-Cabrera M, Letovsky J, Hu KQ, Siddiqui A. Transcriptional factor C/EBP binds to and transactivates the enhancer element II of the hepatitis B virus. Virology. 1991;183(2):825–9. Epub 1991/08/01. . 1853580 10.1016/0042-6822(91)91019-d

[32] Lin SJ, Shu PY, Chang C, Ng AK, Hu CP. IL-4 suppresses the expression and the replication of hepatitis B virus in the hepatocellular carcinoma cell line Hep3B. Journal of immunology. 2003;171(9):4708–16. . 14568946 10.4049/jimmunol.171.9.4708

[33] Ott M TS, Gupta S. Phyllanthus amarus suppresses hepatitis B virus by interrupting interactions between HBV enhancer I and cellular transcription factors. Eur J Clin Invest. 1997;27(11):908–15. 9395786 10.1046/j.1365-2362.1997.2020749.x

[34] Timchenko NA, Harris TE, Wilde M, Bilyeu TA, Burgess-Beusse BL, Finegold MJ, et al CCAAT/enhancer binding protein alpha regulates p21 protein and hepatocyte proliferation in newborn mice. Molecular and cellular biology. 1997;17(12):7353–61. 9372966 10.1128/mcb.17.12.7353 PMC232591

[35] Cha MY, Ryu DK, Jung HS, Chang HE, Ryu WS. Stimulation of hepatitis B virus genome replication by HBx is linked to both nuclear and cytoplasmic HBx expression. The Journal of general virology. 2009;90(Pt 4):978–86. Epub 2009/03/07. 10.1099/vir.0.009928-0 . 19264639

[36] McClain SL, Clippinger AJ, Lizzano R, Bouchard MJ. Hepatitis B virus replication is associated with an HBx-dependent mitochondrion-regulated increase in cytosolic calcium levels. J Virol. 2007;81(21):12061–5. Epub 2007/08/19. 10.1128/JVI.00740-07 17699583 PMC2168786

[37] Park US, Park SK, Lee YI, Park JG, Lee YI. Hepatitis B virus-X protein upregulates the expression of p21waf1/cip1 and prolongs G1—>S transition via a p53-independent pathway in human hepatoma cells. Oncogene. 2000;19(30):3384–94. 10.1038/sj.onc.1203674 . 10918595

[38] Qiao L, Leach K, McKinstry R, Gilfor D, Yacoub A, Park JS, et al Hepatitis B virus X protein increases expression of p21(Cip-1/WAF1/MDA6) and p27(Kip-1) in primary mouse hepatocytes, leading to reduced cell cycle progression. Hepatology. 2001;34(5):906–17. Epub 2001/10/27. 10.1053/jhep.2001.28886 . 11679961

[39] Choi BH PG, Rho HM. Interaction of hepatitis B viral X protein and CCAAT/ enhancer-binding protein alpha synergistically activates the hepatitis B viral enhancer II/pregenomic promoter. J Biol Chem. 1999;274(5):2858–65. 9915821 10.1074/jbc.274.5.2858

